# Effects of *Portulaca oleracea* (purslane) on liver function tests, metabolic profile, oxidative stress and inflammatory biomarkers in patients with non-alcoholic fatty liver disease: a randomized, double-blind clinical trial

**DOI:** 10.3389/fnut.2024.1371137

**Published:** 2024-07-29

**Authors:** Narges Milkarizi, Hanieh Barghchi, Saba Belyani, Hossein Bahari, Farnood Rajabzade, Andisheh Norouzian Ostad, Ladan Goshayeshi, Mohsen Nematy, Vahid Reza Askari

**Affiliations:** ^1^Department of Nutrition, Faculty of Medicine, Mashhad University of Medical Sciences, Mashhad, Iran; ^2^Student Research Committee, Mashhad University of Medical Sciences, Mashhad, Iran; ^3^Student Research Committee, North Khorasan University of Medical Sciences, Bojnourd, Iran; ^4^Transplant Research Center, Clinical Research Institute, Mashhad University of Medical Sciences, Mashhad, Iran; ^5^Department of Radiology, Mashhad Medical Sciences Branch, Islamic Azad University, Mashhad, Iran; ^6^Department of Gastroenterology and Hepatology, Faculty of Medicine, Mashhad University of Medical Sciences, Mashhad, Iran; ^7^Gastroenterology and Hepatology Research Center, Mashhad University of Medical Sciences, Mashhad, Iran; ^8^Metabolic Syndrome Research Center, Mashhad University of Medical Sciences, Mashhad, Iran; ^9^International UNESCO Center for Health-Related Basic Sciences and Human Nutrition, Mashhad University of Medical Sciences, Mashhad, Iran; ^10^Pharmacological Research Center of Medicinal Plants, Mashhad University of Medical Sciences, Mashhad, Iran

**Keywords:** non-alcoholic fatty liver, liver steatosis, *Portulaca oleracea*, oxidative stress, inflammation, purslane

## Abstract

**Background:**

Non-alcoholic fatty liver disease (NAFLD) is a prevalent chronic liver disease. *Portulaca oleracea* exhibits anti-oxidant, anti-inflammatory, and hepatoprotective effects. This clinical trial aimed to investigate the potential benefits of *Portulaca oleracea* in improving NAFLD.

**Methods:**

This double-blind, randomized clinical trial enrolled 70 patients with NAFLD assigned to either the intervention group (*n* = 35) or placebo group (*n* = 35) using stratified block randomization. The intervention group received 700 mg *Portulaca oleracea* supplement for eight weeks, while the control group received placebo capsules. In addition, all participants received a calorie-restricted diet. Liver steatosis and fibrosis were assessed using elastography along with liver function and metabolic tests, blood pressure measurements, body composition analysis and dietary records pre-and post-intervention.

**Results:**

The average age of the participants was 44.01 ± 8.6 years, of which 34 (48.6%) were women. The group receiving *Portulaca oleracea* showed significant weight changes, body mass index, fat mass index, and waist circumference compared to the placebo (*p* < 0.001). In addition, blood sugar, lipid profile, liver enzymes aspartate and alanine transaminase, gamma-glutamyl transferase, and systolic blood pressure were significantly improved in the intervention group compared to those in the placebo (*p* < 0.05). During the study, inflammatory and oxidative stress indicators, improved significantly (*p* < 0.05). Based on the elastography results, the hepatorenal ultrasound index and liver stiffness decreased significantly in the *Portulaca oleracea* group compared to the placebo (*p* < 0.001).

**Conclusion:**

The present clinical trial showed that receiving *Portulaca oleracea* supplement for eight weeks can improve the condition of liver steatosis and fibrosis in patients with NAFLD.

## 1 Introduction

Nonalcoholic fatty liver disease (NAFLD) is the most common cause of chronic liver disease worldwide. The global prevalence of this disease is estimated to be between 25 and 40% in high-risk groups and is rapidly increasing (1). The prevalence of NAFLD has increased in recent years along with the obesity epidemic, type 2 diabetes, hypertension, and hypercholesterolemia (2). NAFLD encompasses a spectrum of liver diseases including steatosis, non-alcoholic steatohepatitis (NASH), fibrosis, cirrhosis, and hepatocellular carcinoma (1).

Approximately 25–40% of NASH patients progress to advanced liver fibrosis; 20–30% of this group will develop cirrhosis and its associated complications. Numerous long-term studies have shown that the mortality rate is 60% in patients with NAFLD with advanced fibrosis and 9% in patients with NAFLD without advanced fibrosis (3).

The management of non-alcoholic fatty liver disease is based on treating the associated liver and metabolic disorders, such as obesity, dyslipidemia, and insulin resistance. Lifestyle modifications, including dietary changes and physical activity aimed at gradual weight loss, are the mainstay of NAFLD management (4, 5), and therapy with the primary goal of improving liver status is recommended only for those with non-alcoholic steatohepatitis or liver fibrosis (6). Interestingly, several recent studies have revealed that dietary components or medicinal plants (with pharmacological capabilities) could be considered substitutes for traditional NAFLD management methods (7–12). Among them, special attention has been paid to supplements that can effectively reduce oxidative stress, which is known to cause hepatic lipotoxicity and inflammation, one of the leading causes of disease pathogenesis (13). However, given the prevalence and complications of the disease, there is still a need for therapeutic supplements to reduce inflammatory status and oxidative stress and improve the lipid profile and nutritional status of these patients (14, 15). *Portulaca oleracea* is an important medicinal plant is *Portulaca oleracea*, that is a rich source of biologically active compounds, including omega-3 fatty acids and β-carotene (16), amino acids, α-tocopherols, ascorbic acid, glutathione (GSH) (17), and flavonoids (18). The anti-inflammatory activity of purslane has been confirmed in several studies (19–24). Hepatoprotective (25–29) and anti-diabetic effects (30, 31) of *Portulaca oleracea* extract have also been reported in animal studies. Therefore, Based on its anti-inflammatory and antioxidant properties as well as its ability to reduce the expression of pro-fibrogenic cytokines and decrease collagenolytic activity, it has been shown that *Portulaca oleracea* has anti-steatotic and anti-fibrotic effects on the liver (32, 33).

Various studies have investigated the effects of *Portulaca oleracea* on different aspects (34). However, to our knowledge, no human study has been conducted on the effect of aerial part extracts on non-alcoholic fatty liver fibrosis. Significant animal studies have been carried out on the aerial parts of *Portulaca oleracea*, demonstrating its beneficial effects on fatty liver in mice, including antioxidant, anti-inflammatory, and liver-protective properties attributed to its alkaloids, flavonoids, terpenoids, sterols, omega-3 unsaturated fatty acids, and numerous vitamin and mineral components (22).

Given the current lack of effective treatments for fatty liver disease and the wide range of pharmacological properties attributed to *Portulaca oleracea* due to its demonstrated anti-inflammatory and antioxidant properties, this study aims to investigate the effects of *Portulaca oleracea* extract supplementation on various aspects of NAFLD, including evaluating its impact on liver steatosis and fibrosis degrees. Secondary objectives involve assessing its effects on dietary intake, weight, body composition, serum lipid profiles, insulin resistance, fasting blood sugar (FBS) levels, inflammatory markers, liver function tests, and oxidative stress levels. We expect that supplementation will lead to improvements in these parameters, potentially reducing the severity of NAFLD. Confirming the efficacy of *Portulaca oleracea* extract in human subjects could introduce a novel therapeutic strategy for NAFLD management, emphasizing the importance of integrating traditional medicine with modern pharmacology for addressing complex diseases effectively.

## 2 Materials and methods

### 2.1 Study design and patient selection

The present study was a randomized, double-blind, parallel, and placebo-controlled clinical trial designed to investigate the effects of a hydroethanolic extract of the aerial parts of *Portulaca oleracea* on clinical and laboratory outcomes in patients with non-alcoholic fatty liver disease. This study was conducted between December 2021 and November 2022 in Mashhad, Razavi Khorasan Province, Iran. The study was conducted according to the principles of the Helsinki Declaration and approved by the ethics committee of the Mashhad University of Medical Sciences (ethics code: IR.MUMS.REC.1400.223). The study protocol have been recently published (35). At the outset of the study, written informed consent was obtained from all participants after providing them with complete information regarding the study procedures.

Participants were recruited from among patients with non-alcoholic fatty liver disease who had been referred to the nutrition clinic and met the inclusion criteria.

Inclusion criteria:

- Participants aged between 18 and 65 years were eligible.
- Diagnosis of steatosis was confirmed via two-dimensional elastography.
- Participants demonstrated willingness to participate in the study.
- Non-alcoholic fatty liver patients classified with F0 and F1 grades were included.

Exclusion criteria:

- Participants who were pregnant or lactating were excluded.
- Individuals with a history of diabetes, autoimmune disorders, cancer, liver failure, viral hepatitis, liver surgery, or kidney disorders (GFR <50) were excluded.
- Participants with a known allergy to *Portulaca oleracea* or herbal supplements were excluded.
- Individuals using hepatotoxic medications such as amiodarone, tamoxifen, sodium valproate, or Methotrexate were excluded.
- Participants with a body mass index (BMI) exceeding 40 were excluded.
- Those with alcohol consumption exceeding 30 g/day for men or 20 g/day for women were excluded.
- Individuals with a history of bariatric surgery were excluded.
- Non-alcoholic fatty liver patients classified with F2, F3, or F4 grades were excluded.
- Participants using multivitamins, antioxidants such as vitamins C and E, silymarin, or herbal medicines regularly were excluded.
- Those who did not comply with weight loss programs in the preceding three months were excluded.

Additionally, participants who became pregnant, developed sensitivity to *P. oleracea* extract or placebo, or exhibited less than 70% compliance with their assigned treatment during the study period were excluded from further analysis.

### 2.2 Randomization and blinding

To minimize potential biases and enhance comparability between study groups, participants were randomly allocated to either the intervention or control groups using a stratified block randomization method. This process ensured balanced distribution across key demographic factors, including age (18–40 years and 40–65 years) and sex (male/female). Randomization was achieved through four blocks in equal proportions.

Participants assigned to the intervention group received two capsules of *Portulaca oleracea* extract daily, each containing 350 mg of hydroethanolic extract derived from the aerial parts of *Portulaca oleracea*. These capsules were administered with meals (breakfast and dinner) for a duration of eight weeks, alongside a prescribed dietary regimen tailored for NAFLD management.

Conversely, participants assigned to the control group received two placebo capsules daily with meals for the same duration of eight weeks. These placebo capsules contained an inert compound, avicel, and were identical in appearance to the *Portulaca oleracea* capsules. Both intervention and control groups followed the same dietary regimen throughout the study period.

To ensure blinding, neither the participants nor the researchers, except for the pharmacist responsible for capsule preparation, were aware of the group assignments until the completion of the study and subsequent data analysis. Blinding was maintained throughout the study in a double-blind manner. Coded containers were utilized to conceal the random allocation of participants to their respective groups, further preserving the integrity of the blinding process.

### 2.3 Procedures

#### 2.3.1 Dose selection

The dosage of *P. oleracea* extract (700 mg daily) was determined based on a previous study in rats (22) and a pilot trial involving five individuals to assess potential challenges or adverse effects.

#### 2.3.2 Preparation of capsules

A 70% hydroalcoholic extract was prepared from the aerial parts of *P. oleracea*. The extract was standardized using the Folin-Miran method, and its components were identified using LC–MS/MS. Capsules containing 350 mg of dried extract and 150 mg of Avicel were prepared for the intervention group, while placebo capsules containing only Avicel and a green colorant were prepared for the control group.

#### 2.3.3 Intervention

Seventy patients with non-alcoholic fatty liver disease were enrolled and randomly assigned to either the intervention or control group. The intervention group received *P. oleracea* capsules (350 mg) twice daily for 60 days, while the control group received placebo capsules. Both groups were provided with a hypocaloric diet and physical activity guidelines.

#### 2.3.4 Follow-up

Participants were followed up through weekly telephone calls and visits during the final week of each 30-day period to assess their well-being and monitor progress.

### 2.4 Data collection and analysis

Data were collected three times: at baseline, on day 30 of the intervention, and on day 60 of the follow-up visit. A questionnaire was used to collect demographic data. Additionally, blood pressure (via a Riester Nova.1032), body mass index, body composition (via a bioimpedance device, “TANITA”), height (via a standard meter), weight (via a portable scale, “Balas”), gastrointestinal complications, and blood pressure were measured at baseline, on the 30th day, and at the end of the intervention period. Transient elastography was performed at the beginning and end of the trial to assess the state of the liver. At baseline, on the 30th day, and at the end of the intervention, participants completed a 3-day food diary and the International Physical Activity Questionnaire (IPAQ). To reduce bias, the responder was asked to clarify any inconsistent answers. Ten milliliters of venous blood was drawn from each patient at the beginning and end of the study to assess biochemical factors such as CBC-diff (using Sysmex KX21), lipid profiles, FBS, serum insulin, hepatic enzymes such as alkaline phosphatase (ALP), aspartate aminotransferase (AST), alanine aminotransferase (ALT), gamma-glutamyl transferase (GGT), total and direct bilirubin (via Auto analyzers), as well as inflammatory and oxidative stress markers such as high-sensitivity C-reactive protein (hs-CRP), erythrocyte sedimentation rate (ESR), Glutathione peroxidases (GPx), and Malondialdehyde (MDA) (using Colorimetry^a^). Serum samples were isolated, and ELISA kits were used to measure the markers.

### 2.5 Study measurements

The primary outcome, liver steatosis, was assessed using the SuperSonic Aixplorer device and a two-dimensional elastography technique at baseline and the end of the study. Secondary outcomes included biochemical evaluations, food intake, anthropometric measurements, blood pressure, physical activity, and other variables. Fasting blood samples were collected at baseline and the end of the study to measure serum levels of liver enzymes, lipid profile, glucose, insulin, and markers of oxidative stress and inflammation. Participants completed a three-day food record at the beginning of the study and at the end of each month to evaluate dietary intake. Height, weight, body composition, and waist circumference were measured at baseline, day 30, and the end of the intervention period. Blood pressure was measured at each visit using a calibrated and standardized mercury sphygmomanometer. The International Physical Activity Questionnaire-Short Form (IPAQ) was used to collect data on participants’ physical activity levels at baseline, day 30, and the end of the intervention period. Demographic and socioeconomic information, as well as gastrointestinal symptoms, were assessed using specific questionnaires.

Detailed information on the methodology, including comprehensive procedures for capsule preparation and intervention, safety considerations, and an in-depth description of the study measurements, is available in the Supplementary materials.

### 2.6 Statistical analyses

In this study, the sample size was determined using the A’Hern RP method and the formula for comparing two proportions related to a qualitative trait, specifically hepatic steatosis, which is applicable to Phase 2 clinical trials (24). Based on the results obtained from treating patients with non-alcoholic fatty liver disease using an anti-oxidant supplement (36), it was assumed that the intervention group would exhibit improvements in hepatic steatosis. With a significance level (alpha) of 0.05, power (beta) of 0.2 (equivalent to 80 percent power), and utilizing the formula for comparing two proportions associated with the qualitative trait of hepatic steatosis, a minimum of 30 participants per group was deemed necessary. The sample size was increased to 36 individuals per group, with a maximum potential dropout rate of 20%.

Following the completion of data collection, all gathered data were entered into the statistical software SPSS version 26 for further analysis. The dataset in this study comprised qualitative and quantitative variables of both continuous and discrete natures. Descriptive and inferential statistical methods were used for data analysis. Before conducting the analyses, the normality of the distribution of quantitative variables was assessed using the Shapiro–Wilk test. Parametric statistical tests were applied to variables that exhibited a normal distribution, whereas non-parametric equivalents were used for variables that did not meet the normality assumption.

Descriptive statistics such as mean, standard deviation, and frequency distribution were used to summarize the demographic information and individual characteristics of the study participants. Quantitative data that followed a normal distribution are presented as mean ± standard deviation, whereas non-normally distributed quantitative data are reported using the median and interquartile range. Qualitative data were summarized using frequency counts and percentages.

To account for missing data, the Last Observation Carried Forward (LOCF) method was employed to impute the missing values using the last observed measurement. The distribution of qualitative variables was examined using the chi-square test for comparisons between and within the groups. Independent sample t-tests were conducted for normally distributed quantitative variables for between-group comparisons and paired t-tests were used for within-group comparisons. In cases where the distribution of quantitative variables was non-normal, non-parametric tests, such as the Mann–Whitney U and Wilcoxon signed-rank tests, were employed.

To control for potential confounding factors such as baseline values, changes in energy intake, physical activity, and body weight, analysis of covariance (ANCOVA) was used. Qualitative variables are presented as frequency counts and percentages. In contrast, quantitative variables with skewness less than one were reported as mean ± standard deviation, and those with skewness greater than or equal to one were presented as median (interquartile range). A statistical significance level of less than 5 percent (*p* < 0.05) was considered statistically significant.

## 3 Results

### 3.1 Characteristics of patients

**Figure 1:**
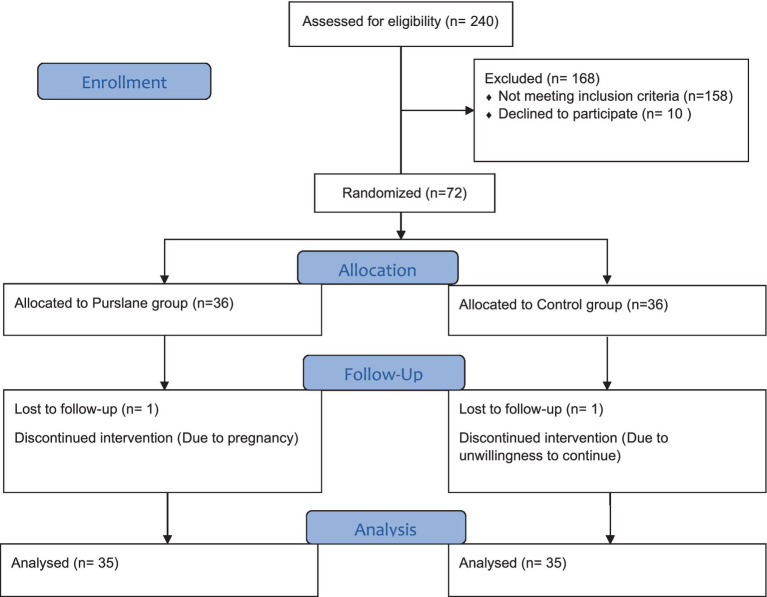
Consort flow diagram.

A total of 240 patients were screened for eligibility and 82 met the inclusion criteria. Ten patients declined to participate in the study, and 72 patients were randomly assigned to the intervention (*n* = 36) and control (*n* = 36) groups. One patient was excluded from each group because of pregnancy or unwillingness to continue the study. Finally, 70 patients (purslane group, *n* = 35; placebo group, *n* = 35) completed the trial (Figure 1).

The baseline patient characteristics are shown in Table 1. The mean age of the patients was 44.01 ± 8.6, of which 51.4% were male. Most of the patients were overweight or obese, and only two patients had a normal BMI. The physical activity level (PAL) of 55.7% of the patients was moderate, and only two had vigorous PAL. There were no significant differences between the purslane and placebo groups in terms of age, sex, BMI, PAL, smoking, and comorbidities, including type 2 diabetes, cardiovascular diseases, hyperlipidemia, and hypertension. Moreover, regarding energy, macronutrient, and micronutrient intake, no significant differences were observed between the two groups (Table 2).

**Table 1:** Baseline characteristics of participants.

Characteristics	Purslane group	Placebo group	*p*-value
Age (years)	43.8 ± 7.6	44.2 ± 9.6	0.85^#^
Weight	85.97 ± 11.36	88.93 ± 13.17	0.31
Height (m)	1.67 ± 1.65	1.76 ± 1.81	0.53
Sex, *n* (%)			
Female	17 (48.6)	17 (48.6)	1.00
Male	18 (51.4)	18 (51.4)	
PAL, *n* (%)			
Mild	13 (37.1)	16 (45.7)	0.76
Moderate	21 (60)	18 (51.4)	
High	1 (2.9)	1 (2.9)	
BMI (kg/m^2^), *n* (%)			
Normal	2 (5.7)	0 (0)	0.22
Overweight	15 (42.9)	20 (57.1)	
Obese	18 (51.4)	15 (42.9)	
Type 2 diabetes, *n* (%)	2 (6.3)	4 (11.4)	0.56
CVD, *n* (%)	2 (6.1)	3 (8.6)	0.71
Hyperlipidemia, *n* (%)	13 (40.6)	11 (31.4)	0.61
Hypertension, *n* (%)	5 (15.2)	6 (17.1)	0.67
Smoking, *n* (%)	3 (8.6)	7 (20)	0.37

Data are presented as Mean ± SD or number (percent). *p* extracted of Chi-Square except (#), which is obtained from two independent sample *t* test. *p* < 0.05 is considered as statistically significant.

**Table 2:** Energy, macronutrient, and micronutrient intake at baseline and at the end of week 8.

Dietary variables	Baseline	After 8 weeks	*p*-value*	Change	*p*-value**
Total energy (kcal)					
Placebo	2524.83 ± 318.82	1899.27 ± 301.46	<0.001	−625.56 ± 131.38	0.55
Purslane	2466.74 ± 294.10	1820.51 ± 264.75	<0.001	−646.23 ± 133.53	
Protein (g)					
Placebo	87.90 ± 18	82 ± 21.10	0.26	−5.80 ± 26.90	0.94
Purslane	89 ± 24.10	82.60 ± 16.50	0.22	−6.40 ± 26.50	
Carbohydrate (g)					
Placebo	352.20 ± 59.30	317.90 ± 61.40	0.01	−34.30 ± 66.30	0.91
Purslane	374.50 ± 52.60	341.50 ± 61.10	0.001	−32.90 ± 37	
Fat (g)					
Placebo	71.09 ± 25.90	66.45 ± 23.30	0.38	−4.64 ± 27.10	0.83
Purslane	73.80 ± 22	67.30 ± 19.70	0.20	−6.50 ± 22.80	
Fiber (g)					
Placebo	18.70 ± 5.70	16.20 ± 6.30	0.12	−2.50 ± 8.30	0.34^&^
Purslane	18.37 ± 4.10	17.70 ± 4.60	0.54	−0.67 ± 5.90	
Cholesterol (mg)					
Placebo	227 ± 105	217 ± 109	0.68	−10 ± 135	0.62
Purslane	236 ± 91	211 ± 103	0.28	−25 ± 118	
SFA (g)					
Placebo	21.87 ± 8.80	19.37 ± 6.80	0.06	−2.5 ± 6.90	0.23
Purslane	22.73 ± 8.70	17.05 ± 8.90	0.002	−5.68 ± 8.4	
Vitamin E (mg)					
Placebo	8.64 ± 7.15	6.70 ± 4.40	0.27^#^	−1.95 ± 8.90	0.78^&^
Purslane	9.96 ± 5.60	7.40 ± 4.10	0.06^#^	−2.56 ± 6.70	

Data are presented as Mean ± SD. *p* * is extracted from Paired *t*-test and *p* ** is extracted from two independent sample *t* test. *p* ^&^ is extracted from Mann–Whitney and *p* ^#^ is extracted from Wilcoxon. *p* < 0.05 is considered as statistically significant.

### 3.2 Anthropometric measurements and blood pressure

Table 3 demonstrates the anthropometric measurements and blood pressure of the participants at baseline and at weeks 4 and 8. Weight, BMI, waist circumference (WC), and fat mass index (FMI) significantly decreased after 8 weeks in the purslane group compared to those in the control group (*p*-value <0.001) and remained significant after adjusting for baseline values (*p*-value <0.001). The control and intervention groups experienced reduced systolic and diastolic blood pressures; however, this reduction was significantly greater in the intervention group after adjusting for baseline values, weight, energy intake, and PAL.

**Table 3:** Anthropometric measurements and blood pressure at baseline and at the end of intervention.

Variables	Baseline	Week 4	Week 8	Change	*p*-value (group)	*p*-value (time)	*p*-value (group/time)	*p*-value^&^
Weight (kg)								
Placebo	88.9 ± 13	87.9 ± 13	87.5 ± 13	−1.4 ± 1.9	0.17	<0.001	<0.001	<0.001
Purslane	85.9 ± 11	84.1 ± 11	82.1 ± 11	−3.8 ± 2.4				
Waist circumference (cm)								
Placebo	108.4 ± 7.9	107.1 ± 8	106.3 ± 8	−2.08 ± 3	0.21	<0.001	<0.001	<0.001
Purslane	107.9 ± 8.9	104.2 ± 9	101.9 ± 9	−5.98 ± 3				
BMI (kg/m^2^)								
Placebo	30.5 ± 4	30.2 ± 4	30 ± 4	−0.52 ± 0.7	0.89	<0.001	<0.001	<0.001
Purslane	30.7 ± 4	30.1 ± 4	29.4 ± 4	−1.37 ± 0.9				
Fat mass index (kg/m^2^)								
Placebo	8.4 ± 3	8 ± 3	8 ± 3	−0.4 ± 0.6	0.60	<0.001	<0.001	<0.001
Purslane	9.1 ± 3	8.5 ± 3	8 ± 3	−1.07 ± 0.7				
Total body water (%)								
Placebo	52.2 ± 6	53.2 ± 6	53 ± 6	0.77 ± 1.7	0.41	<0.001	0.02	0.05
Purslane	50.7 ± 5	51.7 ± 5	52.4 ± 5	1.69 ± 1.4				
Fat-free mass (kg)								
Placebo	64.4 ± 10	64.7 ± 11	64.2 ± 10	−0.24 ± 1.5	0.14	0.02	0.36	0.12
Purslane	61 ± 10	60.7 ± 10	60.4 ± 10	−0.56 ± 1.4				
Trunk fat (%)								
Placebo	25.7 ± 7	24.3 ± 8	24.7 ± 7	−0.98 ± 2.6	0.86	<0.001	0.015	0.06
Purslane	26.5 ± 5	25.1 ± 5	23.9 ± 6	−2.57 ± 2.6				
Systolic blood pressure (mmHg)								
Placebo	136.8 ± 15	134 ± 13	130.5 ± 13	−6.25 ± 9.5	0.24	<0.001	0.064	0.03
Purslane	135.8 ± 13	130.1 ± 11	125.4 ± 11	−10.27 ± 10				
Diastolic blood pressure (mmHg)								
Placebo	87.3 ± 8	84.6 ± 7	82.8 ± 7	−4.45 ± 6.6	0.052	<0.001	0.04	0.01
Purslane	85.9 ± 8	81.5 ± 7	77.7 ± 6	−8.2 ± 8.4				

Data are presented as Mean ± SD. *p* is extracted from Repeated Measures. *p* ^&^ is presented adjusted model by weight and physical activity with ANCOVA. *p* < 0.05 is considered as statistically significant.

### 3.3 Liver function and glycemic indices

**Figure 2:**
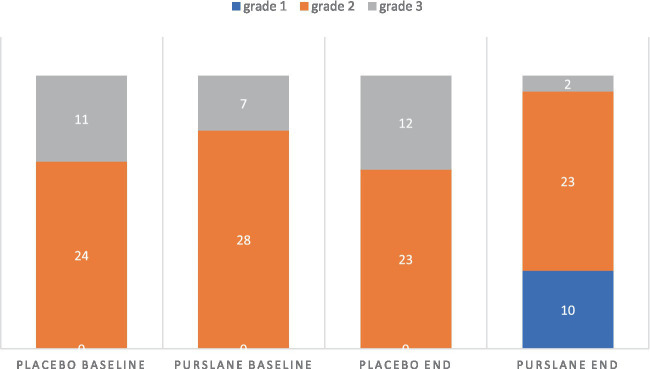
The grade of liver steatosis at baseline and at the end of intervention in placebo and purslane groups.

The changes in liver function and glycemic indices of the two groups after 8 weeks are shown in Table 4. Although there were no significant changes in ALP levels in the two groups, the levels of AST, ALT, GGT, and total and direct bilirubin significantly decreased in the purslane group compared to those in the placebo group. Regarding the glycemic indices, a significant decrease was observed in FBS levels and TyG index after treatment with purslane for 8 weeks. The grades of liver steatosis at baseline and at the end of the intervention are shown in Figure 2. A significant improvement in liver steatosis was observed in the purslane group compared with that in the placebo group.

**Table 4:** Glycemic indices and liver function at baseline and at week 8.

Variables	Baseline	Week 8	Change	*p*-value*	*p*-value^#^	*p*-value^&^
FBS (mg/dl)						
Placebo	103.4 ± 20	103.8 ± 20	0.34 ± 7	0.78	<0.001	<0.001
Purslane	98.4 ± 16	92.9 ± 16	−5.54 ± 6	<0.001		
Insulin (uIU/ml)						
Placebo	19.42 ± 11.5	19.63 ± 11.5	0.21 ± 1.8	0.50	0.22	0.37
Purslane	18.6 ± 9.9	16.86 ± 9.5	−1.74 ± 9.3	0.27		
HOMA-IR						
Placebo	5.05 ± 3	5.78 ± 4.7	0.72 ± 4	0.29	0.08	0.13
Purslane	4.59 ± 3	3.87 ± 2.3	−0.71 ± 2.6	0.12		
TyG index						
Placebo	8.5 ± 0.5	8.49 ± 0.5	−0.01 ± 0.1	0.64	<0.001	<0.001
Purslane	8.79 ± 0.5	8.61 ± 0.5	−0.18 ± 0.1	<0.001		
ALP (U/L)						
Placebo	186.7 ± 42.5	187.4 ± 41.3	0.77 ± 5.5	0.41	0.21	0.29
Purslane	190 ± 41.6	188.4 ± 37.8	−1.6 ± 12	0.33		
AST (U/L)						
Placebo	27.37 ± 14.6	26.57 ± 14.3	−0.8 ± 1.7	0.01	<0.001	<0.001
Purslane	26.66 ± 10.6	20.9 ± 7.5	−5.74 ± 4.5	<0.001		
ALT (U/L)						
Placebo	31.11 ± 16.5	31.4 ± 17	0.28 ± 5.2	0.74	<0.001	<0.001
Purslane	32.11 ± 14.1	25.1 ± 10.8	−7.02 ± 5.9	<0.001		
GGT (U/L)						
Placebo	34.57 ± 12.9	33.88 ± 12.8	−0.68 ± 2.8	0.15	0.002	<0.001
Purslane	32 ± 14	27.65 ± 10.9	−4.34 ± 5.9	<0.001		
AST/ALT						
Placebo	0.91 ± 0.2	0.87 ± 0.2	−0.04 ± 0.1	0.04	0.06	0.07
Purslane	0.87 ± 0.2	0.88 ± 0.2	0.01 ± 0.1	0.62		
Total bilirubin (mg/dl)						
Placebo	1.23 ± 0.3	1.21 ± 0.3	−0.01 ± 0.1	0.28	<0.001	<0.001
Purslane	1.23 ± 0.4	0.99 ± 0.3	−0.23 ± 0.2	<0.001		
Direct bilirubin (mg/dl)						
Placebo	0.25 ± 0.1	0.29 ± 0.2	0.03 ± 0.2	0.32	0.01	0.03
Purslane	0.3 ± 0.1	0.24 ± 0.05	−0.06 ± 0.1	<0.001		
Liver stiffness (kPa)						
Placebo	5.11 ± 0.56	5.26 ± 0.55	0.15 ± 0.21	<0.001	<0.001	<0.001
Purslane	5.36 ± 0.45	4.68 ± 0.55	−0.68 ± 0.36	<0.001		
Hepatorenal Index						
Placebo	1.83 ± 0.25	1.88 ± 0.26	0.05 ± 0.15	0.055	<0.001	<0.001
Purslane	1.88 ± 0.25	1.58 ± 0.2	−0.3 ± 0.18	<0.001		

Data are presented as Mean ± SD. *p* * is extracted from Paired *t*-test and *p* # is extracted from 2 independent sample *t* test. *p* ^&^ is presented adjusted model by weight and physical activity with ANCOVA. *p* < 0.05 is considered as statistically significant.

### 3.4 Lipid profile, inflammation, and oxidative stress

Table 5 illustrates the patients’ lipid profile changes, oxidative stress, and inflammatory markers. There was a significant improvement in the total cholesterol (TC), triglycerides (TG), and high-density lipoproteins (HDL) levels in the purslane group compared to those in the placebo group. In contrast, no significant change was observed in low-density lipoproteins (LDL) levels (*p*-value =0.05). In terms of oxidative stress, MDA levels did not change significantly after the intervention; however, a significant increase in superoxide dismutase (SOD), GPxs, and catalase levels was observed in the intervention group. Regarding inflammatory markers, purslane supplementation significantly decreased IL-6 and hs-CRP levels and ESR (*p*-value <0.001) (see Table 6).

**Table 5:** lipid profile, oxidative stress, and inflammatory markers at baseline and at week 8.

Variables	Baseline	Week 8	Change	*p*-value*	*p*-value^#^	*p*-value^&^
TC (mg/dl)						
Placebo	173.49 ± 23.1	176.89 ± 21.3	3.4 ± 9.8	0.05	< 0.001	< 0.001
Purslane	183.54 ± 35.4	171.86 ± 28.6	−11.68 ± 17	< 0.001		
TG (mg/dl)						
Placebo	107.37 ± 50.2	107.66 ± 52.5	0.28 ± 9.9	0.86	< 0.001	0.005
Purslane	149.43 ± 60.8	131.83 ± 56.4	−17.6 ± 21.9	< 0.001		
HDL (mg/dl)						
Placebo	41.77 ± 5.3	42.54 ± 5.3	0.77 ± 1.8	0.02	< 0.001	< 0.001
Purslane	42.6 ± 6.7	46.63 ± 6.9	4.02 ± 4.5	< 0.001		
LDL (mg/dl)						
Placebo	89.66 ± 12.9	91.29 ± 13	1.62 ± 6	0.12	0.05	0.06
Purslane	92.26 ± 18.3	88.74 ± 14.2	−3.51 ± 8.3	0.18		
MDA (nmol/ml)						
Placebo	0.83 ± 0.19	0.86 ± 0.2	0.03 ± 0.11	0.07	0.051	0.062
Purslane	0.89 ± 0.23	0.81 ± 0.25	−0.08 ± 0.23	0.05		
SOD (U/ml)						
Placebo	235.34 ± 9.3	224.03 ± 10.3	−11.3 ± 9	< 0.001	< 0.001	< 0.001
Purslane	227.45 ± 14	244.94 ± 11	17.48 ± 14.8	< 0.001		
GPx (U/ml)						
Placebo	29.01 ± 6.8	27.69 ± 6.5	−1.31 ± 3.5	0.03	< 0.001	< 0.001
Purslane	26.96 ± 7	33.51 ± 8.1	6.54 ± 3.9	< 0.001		
Catalase (U/ml)						
Placebo	4.21 ± 1.25	3.62 ± 1.35	−0.59 ± 0.54	< 0.001	< 0.001	< 0.001
Purslane	3.76 ± 1.14	6.07 ± 1.39	2.3 ± 0.78	< 0.001		
IL-6 (pg/ml)						
Placebo	12 ± 2.06	12.54 ± 2.1	0.53 ± 1.89	0.10	< 0.001	< 0.001
Purslane	12.95 ± 1.7	10.94 ± 1.8	−2 ± 1.68	< 0.001		
hs-CRP (mg/L)						
Placebo	2.6 ± 1.09	2.9 ± 1.09	0.29 ± 0.42	0.05	< 0.001	< 0.001
Purslane	3.13 ± 2.05	1.93 ± 1.13	−1.19 ± 1.64	< 0.001		
ESR 1 h						
Placebo	14.69 ± 8.2	13.51 ± 6.23	−1.17 ± 3.4	0.05	< 0.001	0.003
Purslane	14.89 ± 8.7	10.46 ± 6.23	−4.42 ± 4.2	< 0.001		
ESR 2 h						
Placebo	24.97 ± 13.14	23.89 ± 21	−1.08 ± 3.89	0.10	< 0.001	< 0.001
Purslane	25.8 ± 12.24	19.8 ± 10.7	−6 ± 4.39	< 0.001		

Data are presented as Mean ± SD. *p* * is extracted from Paired *t*-test and *p* # is extracted from 2 independent sample *t* test. *p* ^&^ is presented adjusted model by weight and physical activity with ANCOVA. *p* < 0.05 is considered as statistically significant.

**Table 6:** Determining the peaks of metabolites in the hydroalcoholic extract of *Portulaca Oleracea* using LC–MS in positive mode.

Peak number	Name of the compound	t_R_ (min)	M + H (*m/z*)	References
1	Portulacanone D	26/9	299/76	(37)
2	Noradrenaline	37/0	170/7	(38)
3	Dopa	15/0	198/12	(39)
4	Oleraceins A	62/5	504/66	(39)
5	Oleraceins B	9/5	533/76	(39)
6	Oleraceins C	64/1	666/06	(39)
7	Oleraceins D	13/1	696/84	(39)
8	Adenosine	19/8	268/8	(39)
9	(3R)-3,5 –Bis (3-methoxy-4-hydroxyphenyl)-2,3 -dihydro-2 (1H)-pyridinone	89/3	342/36	(40)
10	Aurantiamide acetate	36/4	445/8	(41)
11	Cyclo (L-tyrosinyl-L-tyrosinyl)	67/7	327/24	(41)
12	Portuloside A	72/2	332/22	(42)
13	Portulene	66/3	337/02	(29)
14	Lupeol	5/66	427/5	(29)
15	(3S)-3 -O-(𝛽-D-Glucopyranosyl)-3,7 -dimethylocta-1,6-dien-3-ol	8/67	318/12	(43)
16	Friedelane	54/9	413/34	(44)
17	Quercetin	39/4	303/18	(45)
18	Myricetin	55/1	318/24	(45)
19	Genistin	65/4	433/20	(46)
20	Indole-3-carboxylic acid	77/8	162/90	(37)
21	Palmitic acid	62/2	256/14	(47)
22	Stearic acid	37/8	285/18	(47)
23	Caffeic acid	65/8	181/08	(48)
24	Riboflavin	35/0	376/62	(49)
25	Vitamin C	28/5	177/00	(50)
26	𝛼-Tocopherol	67/1	431/22	(47)
27	Hesperidin	76/8	611/58	(50)
28	Portulacerebroside A	64/6	843/18	(44)
29	𝛽-Sitosterol	48/7	415/32	(29)
30	𝛽-Carotene	37/5	538/74	(47)

## 4 Discussion

The findings of our study showed that supplementation with 700 mg of purslane aerial part extract along with a restricted-calorie diet for eight weeks led to an improvement in liver steatosis and liver function indicators (AST, ALT, GGT, and total and direct bilirubin) in patients with NAFLD. In addition, inflammatory factors (IL-6, hs-CRP, and ESR) and oxidative stress factors (SOD, GPx, and CAT) improved significantly. On the other hand, anthropometric indices such as weight, BMI, FMI, WC, metabolic indices (FBS, TyG, TG, HDL, TC), and DBP were also recovered in the purslane recipient group.

In contrast with intervention group, in patients receiving placebo, a slight increase in liver stiffness and hepatorenal index was observed, which was statistically significant. It is possible that nonadherence to the diet and physical activity recommended in the plan among some people in the placebo group could be the reason for this difference.

El-Sayed et al. (51) investigated the antidiabetic activity of purslane seeds in patients with type 2 diabetes. They observed that treatment with 10 g of purslane seed powder for eight weeks significantly decreased total and direct bilirubin, ALT, AST, GGT, TG, TC, LDL-c, FBS, insulin, body weight, and BMI and increased albumin and HDL-c levels. The control group (receiving 1,500 mg of metformin) showed the same results as the purslane group, except for albumin, direct bilirubin, HDL-c, and TG levels. These data show that purslane seeds improve liver function in patients with diabetes compared to metformin. In addition, purslane seeds have been shown to reduce blood glucose, insulin resistance, blood lipids, liver protection, and weight loss due to the content of polyunsaturated fatty acids (PUFA), flavonoids, polysaccharides, antioxidants, and vitamins (52). Various studies have suggested that purslane can significantly reduce the severity of non-alcoholic fatty liver steatosis owing to its hypoglycemic and hypolipidemic properties (53–55). Studies have also shown that obesity and related hormonal changes, including increased insulin synthesis, peripheral insulin resistance, increased leptin synthesis, and decreased adiponectin synthesis by adipose tissue, lead to a decrease in fatty acid oxidation and endothelial dysfunction (56), followed by an increase in blood sugar (57). Increased fasting blood glucose is independently associated with decreased adiponectin levels, which plays a significant role in reducing insulin resistance (58). Purslane can effectively improve liver steatosis through weight loss, increased adiponectin levels, increased fatty acid oxidation, and reduced insulin resistance (59). Ranaei et al. (60) investigated the effects of 400 mg/kg purslane extract on sirtuin levels and insulin resistance in rats with NAFLD. After eight weeks, purslane extract improved insulin resistance in NAFLD by increasing sirtuin levels. Sirtuin levels are associated with insulin resistance, which can predispose patients to the development of NAFLD (61). In addition, the activation of sirtuin by the polyphenols in purslane acts as an upstream regulator in the LKB1/AMPK signaling axis, suppressing Acetyl-CoA Carboxylase (ACC) and Fatty Acid Synthase (FAS) expression and reducing fat accumulation in liver cells (62, 63).

Damavandi et al. (59) investigated the effects of purslane extract in patients with NAFLD. Purslane extract at a dose of 300 mg daily for 12 weeks significantly decreased the ALT, AST, GGT, FBS, HOMA-IR, TG, and LDL-c levels in the purslane group. However, in contrast to our study, at the end of the study, no significant changes were observed in the grade of hepatic steatosis, insulin, liver enzymes, total bilirubin, lipid profile, and blood pressure compared to the control group.

A study by Wainstein et al. (64) reported that purslane extract (three capsules: 180 mg/day) for 12 weeks reduced BMI and body weight in patients with type 2 diabetes. Purslane is known to affect metabolism and body weight (54). In this regard, and in line with our study, the study by Esmaillzadeh et al. (65) showed that a daily intake of 10 g of purslane seeds for five weeks could improve anthropometric indicators (weight, BMI, and waist circumference).

In the present study, purslane supplementation significantly improved SOD levels. We also observed a significant increase in the levels of catalase (CAT) and GPx in the purslane group. In line with our results, in a study conducted by Sousou et al. (32) on rats with liver fibrosis, supplementation with purslane aerial part extract at a dose of 400 mg/kg per day caused a significant decrease in AST, ALT, ALP, GGT, total bilirubin, MDA, and tumor necrosis factor α (TNF-α), and a significant increase in SOD, CAT, GPx, and GSH levels. In this study, we investigated the anti-oxidant effects of purslane hydroethanolic extract against biliary obstruction caused by liver fibrosis in rats and observed that this compound inhibited oxidative stress, reduced the expression of profibrogenic cytokines, collagenolytic activity, and activated hepatic stellate cells.

Purslane, known for its hypolipidemic effects, influences liver function, inflammation, and oxidative stress through various mechanisms. Fresh purslane upregulates cholesterol 7a-hydroxylase (CYP7A1) and low-density lipoprotein receptor (Ldlr) to combat the harmful effects of a high-fat diet on the liver (66). Moreover, purslane’s bioactive compounds, such as omega-3 fatty acids and antioxidants, may improve liver function, reduce inflammation, and combat oxidative stress through enhancing antioxidant enzymes like catalase and glutathione peroxidase, as shown in rat studies (67). Additionally, *Portulaca oleracea* L. flavone (POL-F) from purslane inhibits inflammatory responses by reducing Nitric oxide (NO) secretion and modulating phosphoinositide 3-kinases (p-PI3K) and p-AKT signaling pathways (68). Purslane seed extracts, particularly the methanol extract, exhibit anti-inflammatory properties by reducing TNF-α and IL-1β production through antioxidant pathways (69).

The results of our study showed that supplementation with 700 mg of purslane aerial part extract along with a restricted calorie diet for eight weeks led to a significant decrease in TC and TG levels and a significant increase in HDL-c levels in the purslane group compared with the placebo group. Sabzghabaee et al. (70) studied the antidyslipidemic effects of purslane in 74 obese adolescents. The results showed that the TG, LDL-C, and TC levels decreased significantly in the purslane group. In addition, the differences in LDL-C and TG parameters between the two groups were statistically significant. Purslane has positive effects on serum lipid profile, which may be attributed to its polyphenolic and antioxidant compounds. The uniqueness of purslane as the richest plant source of omega-3 PUFA has been well established. Purslane also contains high amounts of vitamins E and C, beta-carotene, and various flavonoids with anti-atherogenic activity (70).

We observed that supplementation with 700 mg of purslane aerial part extract, along with a restricted calorie diet for eight weeks, led to a significant reduction in DBP. High blood pressure is a known risk factor for NAFLD (71). Consistent with our study, Esmaillzadeh et al. (65) have shown the effects of purslane seeds on lowering blood pressure by receiving 10 g of purslane seeds daily for five weeks. Purslane contains PUFA and omega-3 fatty acids, vitamin E, vitamin C, beta-carotene, alkaloids, flavonoids, and polysaccharides (70). Purslane is the richest source of omega-3, and 100 g of fresh purslane leaves (one serving) contain approximately 300–400 mg of omega-3 (17). Evidence confirms the positive relationship between omega-3 consumption and blood pressure control (72).

### 4.1 Strength and limitations

In the current study, several items can be mentioned as the strengths of this research, which are as follows: In this research, a stratified block design was used to randomly assign the participants to the studied groups. This design resulted in a homogeneous distribution of characteristics between the study groups and the control of confounders. Moreover, to correctly estimate the effect of the intervention, detailed evaluations of potential confounders were made during the study, and their effects were adjusted in statistical models. In this study, the two-dimensional elastography method, which is more accurate than ultrasound and other conventional methods, was used to evaluate liver fibrosis and steatosis.

### 4.2 Future work

Given the promising results observed, future research endeavors should aim to conduct longitudinal studies that extend beyond the 8-week period to assess the durability of treatment effects provided by *Portulaca oleracea* extract on liver health in patients with NAFLD. It would be valuable to investigate the interactions between purslane extract and conventional NAFLD treatments to explore potential synergistic effects. Furthermore, identifying and isolating specific bioactive compounds within purslane extract responsible for its hepatoprotective properties could pave the way for the development of new therapeutic agents. Expanding the participant pool to include a wider range of ethnicities and backgrounds would enhance the external validity of the findings. Moreover, given the complexity of NAFLD as a disease that intersects with metabolic syndrome, future studies might also benefit from exploring the impact of purslane extract across different facets of metabolic health.

## 5 Conclusion

The collective findings from these studies underscore the therapeutic potential of *Portulaca oleracea*, commonly known as purslane, in managing non-alcoholic fatty liver disease (NAFLD) and improving metabolic health. Purslane supplementation was shown to significantly enhance liver function, reduce inflammatory and oxidative stress markers, and improve metabolic parameters such as lipid profiles and blood glucose levels. These beneficial effects are attributed to purslane’s rich composition of bioactive compounds, including antioxidants, omega-3 fatty acids, and various phytochemicals, which collectively contribute to its hepatoprotective, anti-inflammatory, and antidiabetic properties. Given the promising outcomes, purslane presents a viable natural therapeutic agent for addressing NAFLD and potentially other metabolic disorders. However, further research is warranted to explore the long-term benefits, optimal dosages, and underlying mechanisms of action of purslane supplementation.

## Data availability statement

The original contributions presented in the study are included in the article/Supplementary material, further inquiries can be directed to the corresponding authors.

## Ethics statement

The studies involving humans were approved by School of Medicine, Mashhad University of Medical Sciences Biomedical Research Ethics Committee (IR.MUMS.REC.1400.223). The studies were conducted in accordance with the local legislation and institutional requirements. The participants provided their written informed consent to participate in this study.

## Author contributions

NM: Conceptualization, Data curation, Formal analysis, Writing – original draft. HaB: Data curation, Writing – original draft. SB: Data curation, Writing – original draft. HoB: Writing – original draft. FR: Data curation, Investigation, Writing – review & editing. AO: Data curation, Investigation, Software, Writing – original draft. LG: Data curation, Methodology, Validation, Writing – review & editing. MN: Conceptualization, Funding acquisition, Investigation, Project administration, Resources, Writing – review & editing. VA: Funding acquisition, Methodology, Project administration, Resources, Supervision, Validation, Visualization, Writing – review & editing.

## Glossary

ALP: alkaline phosphatase
ALT: alanine aminotransferase
AST: aspartate aminotransferase
BMI: body mass index
CBC-diff: complete blood count with differential count
CRP: C-reactive protein
FBS: fasting blood sugar
GPx: glutathione peroxidase
HbA1c: hemoglobin A1c
IL: interleukin
IPAQ: international physical activity questionnaire
MDA: malondialdehyde
NAFLD: non-alcoholic fatty liver disease
NASH: non-alcoholic steatohepatitis
NF-κβ: nuclear factor kappa β
PI3K: phosphoinositide 3-kinases
RCT: randomized clinical trial
SOD: super oxide dismutase
TNF-ɑ: tumor necrosis factor ɑ
ESR: erythrocyte sedimentation rate
GGT: gamma-glutamyl transferase
TG: triglycerides
TC: total cholesterol
LDL: low-density lipoproteins
HDL: high-density lipoproteins
