# Supplementary material for: Effects of Portulaca oleracea (purslane) on liver function tests, metabolic profile, oxidative stress and inflammatory biomarkers in patients with non-alcoholic fatty liver disease: a randomized, double-blind clinical trial
Source: Front Nutr. 2024 Jul 29;11:1371137. doi: 10.3389/fnut.2024.1371137 (PMC11317426; doi:10.3389/fnut.2024.1371137)
Supplement: Supplementary file 1 [file Presentation_1.pdf]

Supplementary materials

Procedures

I) Dose selection

In this study, the dosage of P. oleracea extract was determined based on a previous investigation conducted by Guoyin et al., where they evaluated the efficacy of P. oleracea supplementation at a dose of 100 mg/kg on liver health in rats under the supervision of a study pharmacologist (1). To extrapolate this dose to human subjects, a conversion factor was applied considering body ratios between humans and the studied animals. The selected dosage of 700 mg was deemed optimal after this conversion process, ensuring a safe and effective dose for human consumption (Figure 1)(1). Additionally, prior to commencing the main study, a pilot trial involving five individuals was conducted to assess any potential challenges or adverse effects associated with the selected dosage. Comprehensive evaluations, including biochemical analyses and digestive assessment questionnaires, were performed, and since no significant issues were reported, the project proceeded with confidence in the selected dosage.

II) Preparation of capsules

Approximately 350 kg of *P. oleracea* plants were collected from Mashhad and thoroughly washed before drying at a low temperature using a dryer. The resulting dried powder was used to create a 70% hydroalcoholic extract. To prepare the extract, 70% edible ethanol, without any preservatives or bitters, was used. The powder was mixed with 70% ethanol and the resulting mixture was placed in a shaker at 37°C for 72–96 h. This step was aimed at separating the active ingredients from the powdered aerial parts of *P. oleracea*. After 96 h, the obtained dark liquid was concentrated using a rotary device and then dried using a freeze-dryer. Once the extract was transformed into a dry powder, its weight was measured, and the efficiency of the extraction process was determined. The total phenol content was measured using the Folin-Miran method to standardize the extract. Additionally, the components of the extracts were identified using LC-MS/MS. To create capsules for administration, with a recommended daily dose of 700 mg, two capsules weighing 500 mg each were used. These capsules consisted of 350 mg dried extract and 150 mg Avicel. The final mixture was comprised of 70% extract and 30% Avicel. *P. oleracea* capsules were prepared using

a capsule filling machine. For the placebo group, which needed to be visually indistinguishable from the intervention group in terms of shape, color, and size, Avicel was used as a filler along with a small amount of green confectionery colorant to match the color of the powder inside the capsules to that of the intervention group. Both placebo and *P. oleracea* capsules were coated with a matte finish to conceal their contents and ensure blinding.

III) Intervention

Seventy patients admitted or transferred to the nutrition clinic in Mashhad, Iran, were enrolled in this study. Prior to their involvement, all the participants provided informed consent to the clinician overseeing the study. Following the randomization process, the intervention group was assigned to receive 60 *P. oleracea* capsules (350 mg) during the initial 30-day period and an additional 60 capsules at the start of the subsequent 30-day period. Participants were instructed to take capsules twice daily, specifically during breakfast and dinner. In contrast, the control group received capsules that were similar in color and quantity following the same prescription. To account for the potential influence of food intake on the outcomes, both the control and intervention groups were provided a hypocaloric diet comprising 55 percent carbohydrates, 30 percent fat, and 15 percent protein. Additionally, participants in both groups received dietary recommendations in accordance with the Mediterranean nutritional guidelines. Furthermore, the researcher provided physical activity guidelines of 150 min per week to all participants.

IV) Follow-up

The follow-up of patients in this study was conducted through the researcher's weekly telephone calls to assess the participants' well-being and monitor their progress. Follow-up visits were scheduled during the final week of each 30-day period.

Safety considerations

Any medical issues or abnormalities identified during the intervention and follow-up procedures were promptly discussed with the participants by a trained medical professional involved in the study. Participants received appropriate treatment, were referred to specialists if necessary, or were advised to schedule appointments with primary healthcare providers. Routine biochemical and clinical assessments were conducted, including liver function tests such as serum levels of alanine aminotransferase (ALT) and aspartate aminotransferase (AST). If any adverse effects or concerns

arose from the intervention, supplementation was immediately discontinued, either temporarily or permanently, based on the medical doctor's discretion.

Study measurements

Two-dimensional elastography

The current study utilized the SuperSonic Aixplorer device (SuperSonic Imagine S.A., Aix-en-Provence, France) and a two-dimensional elastography technique to image liver tissue. Following the methodology outlined in Webb et al., we determined that the numerical values of the hepatorenal sonographic index were 49.1, 86.1, and 23.2 for mild (grade 1), moderate (grade 2), and severe (grade 3) steatosis, respectively (2).

Biochemical evaluations

At baseline and the end of the study, 10 ml fasting blood samples were collected from all participants for biochemical tests by a reference laboratory (Mavid Laboratory). Serum was isolated from blood samples by centrifugation at 3000 rpm for 15 min and immediately stored at -20°C until analysis. Serum levels of alanine aminotransferase, aspartate aminotransferase, gamma-glutamyl transferase, alkaline phosphatase, total and direct bilirubin, high-sensitivity C-reactive protein, fasting glucose, triglycerides, total cholesterol, high-density lipoprotein, and low-density lipoprotein were measured using colorimetric assays on an Alpha Classic AT Plus analyzer.

Insulin levels were measured using a fluorescence-based kit (Snibe, Shenzhen, China) and a Maglumi 800 device. Insulin resistance was assessed using the homeostatic model assessment of insulin resistance (HOMA-IR) and fasting triglyceride glucose index. Additionally, the total cholesterol/HDL cholesterol ratio, a measure of cardiovascular disease risk, was calculated at baseline and at the end of the study period.

Concurrently with blood collection from the participants, 1 ml of serum was stored at -80°C until analysis for markers of oxidative stress (malondialdehyde, glutathione peroxidase, superoxide dismutase, and catalase) and inflammation (hs-CRP, interleukin-6) before and after the intervention. Serum samples were stored in a freezer at the central laboratory of the Medical School and analyzed using Betagen kits and standard protocols in a single run.

Assessment of food intake

In this study, we utilized a three-day food record, consisting of two weekdays and one weekend day, to evaluate the dietary intake. To ensure an accurate assessment of participants' dietary patterns, we asked them to complete food records at the beginning of the study and at the end of each month. The nutrient and energy intake of each participant was then computed using the Nutritionist IV software (N-Squared Computing, Salem, OR, USA).

Anthropometric assessment

In this study, height and weight were measured under standard conditions(3), without shoes and with minimal clothing, to calculate participants' body mass index (BMI). Height was measured using a wall-mounted stadiometer (Seca 206, Germany) with an accuracy of 0.1 cm, and weight was measured using a digital floor scale (Seca 813, Germany) with an accuracy of 0.1 kg. Body composition was measured using a Tanita BC-418 body composition analyzer (Tanita Corporation, Tokyo, Japan). Waist circumference was measured at the midpoint between the lower border of the rib cage and iliac crest with a flexible, non-stretchable tape measure while the participants were standing with their feet close together and without any pressure on the body surface. The measurement was performed with an approximate accuracy of 1.0 cm(3).

Blood pressure assessment

In this study, the participants' blood pressure was measured at each visit using a calibrated and standardized mercury sphygmomanometer (ALPK2) with a standard cuff size of 12×25 cm. Blood pressure was measured using a digital medical device as a secondary measurement method.

Assessment of physical activity

We used the International Physical Activity Questionnaire-Short Form (IPAQ) to collect the participants' physical activity level data (4). The validity and reliability of the Persian version of this questionnaire has been verified (5, 6). This questionnaire is designed for individuals aged 18 to 65 years and includes questions about the duration and intensity of physical activity in the past seven days. It assesses the hours spent in physical activity at various intensities, including walking and moderate-intensity and vigorous-intensity activities. Each Metabolic Equivalent Task (MET) per week shows the amount of energy spent on physical activity over the week. The calculated MET minutes per week for each type of activity was established as follows:

1. Walking MET-minute/week = 3.3 x walking minutes x walking days.

2. Moderate MET-minute/week = 4.0 x moderate intensity activity minutes x moderate activity days.

3. Vigorous MET-minute/week = 8.0 x vigorous intensity activity minutes x vigorous activity days.

The total MET-min/week value was calculated by adding the walking, moderate, and vigorous MET-minute/week values. Participants were classified as having low, moderate, or high levels of physical activity based on their total MET minutes per week, with less than 600 MET minutes per week classified as low activity, 600 to 3000 MET minutes per week classified as moderate activity, and more than 3000 MET minutes per week classified as high activity.

Evaluation of other variables

Demographic and socioeconomic information on individuals was obtained using a specific questionnaire. The questionnaire included questions on education level, family size, housing status, occupation, medical history, and use of medications and supplements. In this study, questions were asked about potential gastrointestinal symptoms, which were evaluated using the Gastrointestinal Symptom Severity Rating (GSSR) questionnaire (7). This questionnaire consisted of 15 questions scored on a Likert scale ranging from no discomfort (zero) to severe discomfort (seven). It assesses five different dimensions: abdominal pain (heartburn, hunger pain, nausea), reflux (stomach burning and food regurgitation), diarrhea (loose stools, urgent need to defecate), constipation (difficulty passing stools, painful defecation, sensation of incomplete evacuation), and dyspepsia (abdominal bloating, belching, stomach rumbling, increased abdominal gas)(7).

LC-MS analysis of purslane hydroalcoholic extract

Liquid chromatography-mass spectrometry (LC-MS) was performed using an AB SCIEX QTRAP liquid chromatography system (Shimadzu) coupled with a triple quadrupole mass spectrometer. Liquid chromatography separation was performed using a Supelco C18 column (15 mm×2.1 mm×3μm). The analysis was performed at a flow rate of 0.2 mL/min. The mobile phase consisted of 0.1% formic acid in water (A) and 0.1% formic acid in acetonitrile (B). The gradient analysis started with 10% B, maintained at isocratic conditions for 10 min, gradually reached 30% B over 20 min, further increased to 80% B over 30 min, held for 10 min, and then returned to the initial conditions of 10% B for 5 min. Finally, the system was equilibrated for 5 minutes. Mass spectra

were acquired in the range of 100–1700 during an 80-minute scan time. Electrospray ionization (ESI) in the positive mode was employed for mass spectrometry. Feature extraction and peak identification were performed using the MZmine software version 2.3.

LC-MS result

Overall, 30 compounds were identified in the hydroalcoholic extract of the aerial parts of *Portulaca oleracea*, primarily comprising alkaloids, flavonoids, terpenoids, and vitamins. Data related to compound identification are presented in Table 6. The total ion chromatograms of the plant extracts are shown in Figure 2A. The MS spectral data were compared with those reported in previous studies. Figures 2B–G represent examples of extracted ion chromatograms from the total ion chromatogram and their corresponding masses. One of the important chemical constituents found in *Portulaca oleracea* are alkaloids, including dopamine and norepinephrine. Additionally, alkaloids such as A, B, C, and D are cyclopdopa alkaloids isolated from the aerial parts of the plant. Furthermore, *Portulaca oleracea* contains monoterpene compounds such as portulacozide A and diterpene compounds such as portulene. It is also rich in ascorbic acid, α-tocopherol, and riboflavin. Therefore, the aerial parts of *Portulaca oleracea* serve as valuable sources of alkaloids, flavonoids, terpenoids, and vitamins.

Figures

Figure 1. Dose conversion of animal study for human trials.

Formula:

Human Equivalent Dose (mg/kg) = Animal dose (mg/kg) × (Animal Km /Human Km)

Calculation:

1) Animal dose =100 mg/kg

2) Animal km =3

3) Human km =37

The human equivalent dose was 8.10 mg/kg .According to the formula mentioned above, the dose of *P. oleracea* plant extract in a 70 kg human is equivalent to 700 mg per day, which was prescribed in 2 doses of 350 mg per day according to the recommendation of the project pharmacologist.

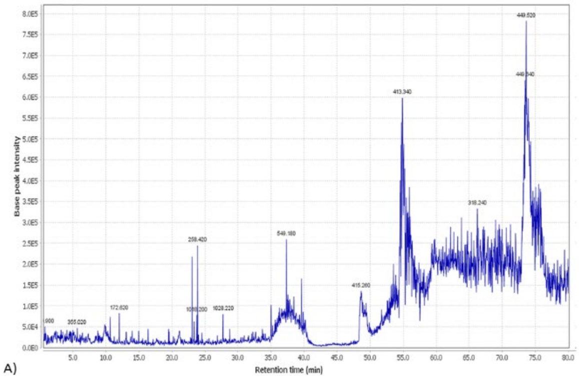
Base peak intensity
Retention time (min)
449.520
449.340
413.340
318.240
549.180
298.400
415.260
172.620
355.020
1028.220
A)

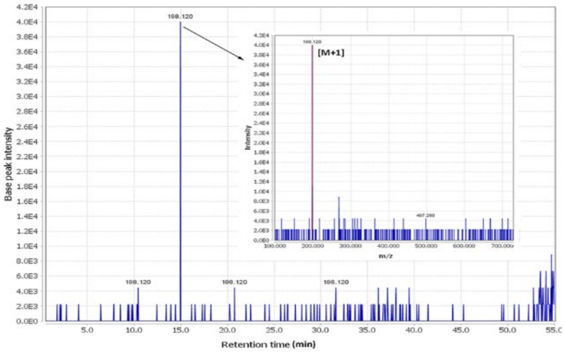
Base peak intensity
Retention time (min)
198.120
[M+1]
Intensity
m/z
B)

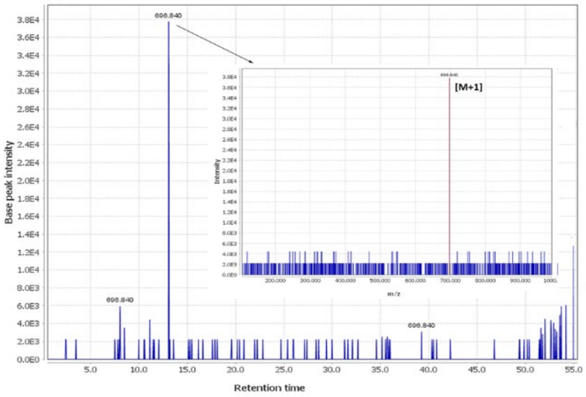
Base peak intensity
Retention time
696.840
[M+1]
Intensity
m/z
C)

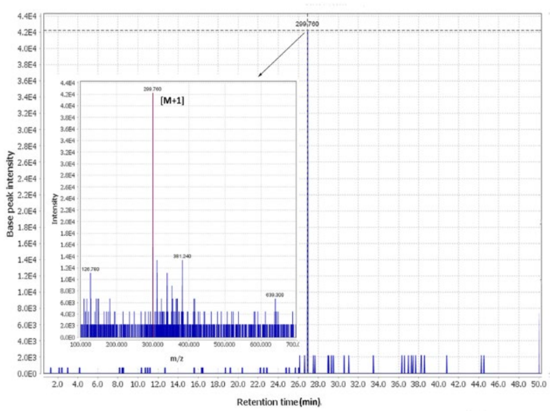
Base peak intensity
Retention time(min)
299.760
[M+1]
Intensity
m/z
D)

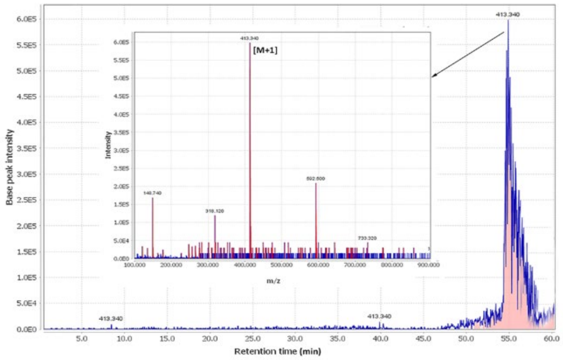
Base peak intensity
Retention time (min)
413.340
[M+1]
Intensity
m/z
E)

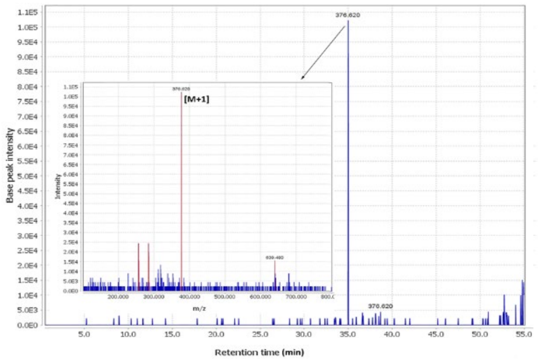
Base peak intensity
Retention time (min)
376.620
[M+1]
Intensity
m/z
F)

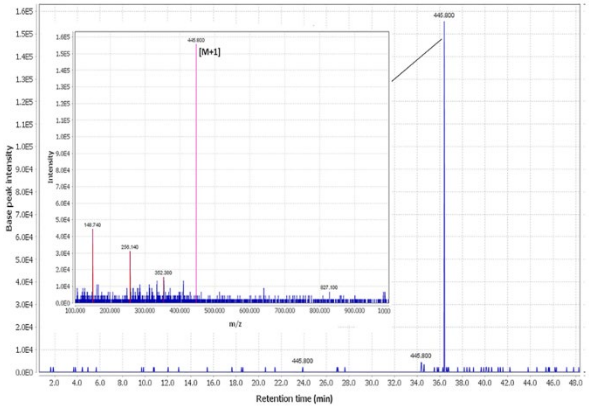
Base peak intensity
Retention time (min)
446.800
[M+1]
Intensity
m/z
G)

Figure 2:

A) Total ion chromatogram of the hydroalcoholic extract of Portulaca oleracea.

B) Chromatogram of dopamine and its corresponding mass spectrum [M+1], at m/z 198/120.

C) Chromatogram of ursine D and its corresponding mass spectrum [M+1], at m/z 696/84.

D) Chromatogram of portulacsonon D and its corresponding mass spectrum [M+1], at m/z 299/76.

E) Chromatogram of feridulan and its corresponding mass spectrum [M+1], at m/z 413/34.

F) Chromatogram of riboflavin and its corresponding mass spectrum [M+1], at m/z 376/62.

G) Chromatogram of orantiamide and its corresponding mass spectrum [M+1], at m/z 445/8.

References

1. Zheng G, Mo F, Ling C, Peng H, Gu W, Li M, et al. Portulaca oleracea L. alleviates liver injury in streptozotocin-induced diabetic mice. Drug Des Devel Ther. 2018;12:47-55.
2. Webb M, Yeshua H, Zelber-Sagi S, Santo E, Brazowski E, Halpern Z, et al. Diagnostic value of a computerized hepatorenal index for sonographic quantification of liver steatosis. AJR American journal of roentgenology. 2009;192(4):909-14.
3. Raymond JL, Morrow K. Krause and Mahan's Food and the Nutrition Care Process: Elsevier; 2020.
4. Craig C, Marshall A, Sjostrom M, Bauman A, Lee P, Macfarlane D, et al. International physical activity questionnaire-short form. J Am Coll Health. 2017;65(7):492-501.
5. Amini H, Isanejad A, Chamani N, Movahedi-Fard F, Salimi F, Moezi M, et al. Physical activity during COVID-19 pandemic in the Iranian population: A brief report. Heliyon. 2020;6(11):e05411.
6. Hazavehei SMM, Asadi Z, Hassanzadeh A, Shekarchizadeh P. Comparing the effect of two methods of presenting physical education Π course on the attitudes and practices of female Students towards regular physical activity in Isfahan University of Medical Sciences. Iranian Journal of Medical Education. 2008;8(1):121-31.
7. Mazaheri M, SadatKhoshouei M. Comparison between Psychometric Characteristics of Persian Version of the Gastrointestinal Symptoms Rating Scale in Functional Gastrointestinal Disorders and Normal Groups. GOVARESH. 2012;17(1):7.
